# Rare giant epithelioid inflammatory myofibroblastic sarcoma of the abdominal cavity in a child: a case report and review of the literature

**DOI:** 10.3389/fonc.2024.1417918

**Published:** 2024-12-05

**Authors:** Jinzhou Li, Haixing Su, Sheng Zhang, Xianyun Chen, Chongzhi Hou, Tao Cheng

**Affiliations:** Department of General Surgery, Xi’an Children’s Hospital/Children’s Hospital Affiliated to Xi’an Jiaotong University, Xi’an, China

**Keywords:** childhood tumor, epithelioid inflammatory myofibroblastic sarcoma, anaplastic lymphoma kinase, crizotinib, targeted therapy

## Abstract

Epithelioid inflammatory myofibroblastic sarcoma (EIMS) is a distinct subtype of inflammatory myofibroblastoma tumor (IMT) that is recognized as a rare malignant tumor characterized by anaplastic lymphoma kinase (ALK) positivity, significant aggressiveness, treatment challenges, and a poor prognosis. We report on the case of an 8-year-old boy presenting with abdominal pain and vomiting. Computed tomography (CT) of the abdomen revealed a large tumor, and the pathology results following a biopsy confirmed the diagnosis of EIMS. The patient underwent radical tumor resection, and genetic testing identified the presence of the *RANBP2*–*ALK* fusion. To our knowledge, this represents the largest pediatric case of abdominal EIMS documented in the literature. Currently, there is no standard therapy for EIMS; however, existing studies advocate for the use of ALK tyrosine kinase inhibitors (TKIs) in its treatment. This case was reported to be in remission following treatment with crizotinib, thereby contributing to the understanding of the specific pathology of EIMS and facilitating accurate diagnosis and targeted therapy.

## Introduction

Inflammatory myofibroblastic tumor (IMT) is a mesenchymal tumor characterized by spindle cells of myofibroblasts embedded in a myxoid or collagenous stroma, which primarily contains plasma cells and lymphocytes, along with occasional eosinophils and neutrophils. The annual incidence of IMT is less than one in a million, with approximately 50%–60% of cases occurring in children and adolescents. IMT is typically located in the abdomen, pelvis, mediastinum, or retroperitoneum. Previously classified as a reactive tumor within the category of “inflammatory pseudotumor,” IMT is now recognized as a distinct tumor with moderate malignant potential. The recurrence rate of IMT ranges from 2% to 25%, while the metastatic rate is reported to be less than 5% (1). Mariño-Enríquez first identified a rare variant of IMT that exhibited specific morphological and immunohistochemical features, which he termed epithelioid inflammatory myofibroblastic sarcoma (EIMS) (2). In contrast to the conventional spindle cell of IMT, EIMS is characterized by an epithelioid morphology and a loose or mucus-like stroma that contains a rich neutrophilic inflammatory infiltrate (3). EIMS can occur across a wide age range, from 7 months to 63 years, frequently involves the abdominal cavity, and is more prevalent in boys/men. Compared with classic IMT, patients with EIMS consistently experience rapid recurrence and have a median overall survival of 11 months (4).

EIMS exhibits positive immunostaining for nuclear membrane or perinuclear anaplastic lymphoma kinase (ALK), and several studies have demonstrated that EIMS displays either a *RANBP2*–*ALK* fusion or a *RANBP1*–*ALK* fusion (5, 6). ALK activation is present in all EIMS cases and in 50% of IMT cases, a finding that supports the tumorigenic origin of EIMS, as ALK proteins are recognized as oncogenic in both hematological and solid tumors. EIMS is more aggressive than IMT, with recurrence and metastasis rates exceeding 80% and 25%, respectively (7, 8). EIMS is significant as a unique variant of IMT as patients with ALK rearrangements may benefit from treatment with targeted ALK inhibitors such as crizotinib (9). However, due to the rarity of EIMS cases, the current systemic treatment regimens and responses to ALK inhibitors remain uncertain.

To the best of our knowledge, the case under study represents the largest reported case of abdominal EIMS in a child, demonstrating significant efficacy against the ALK inhibitor crizotinib. Given its extreme rarity, this clinical case is thoroughly analyzed, and previous literature reports are summarized.

## Case report

**Figure 1:**
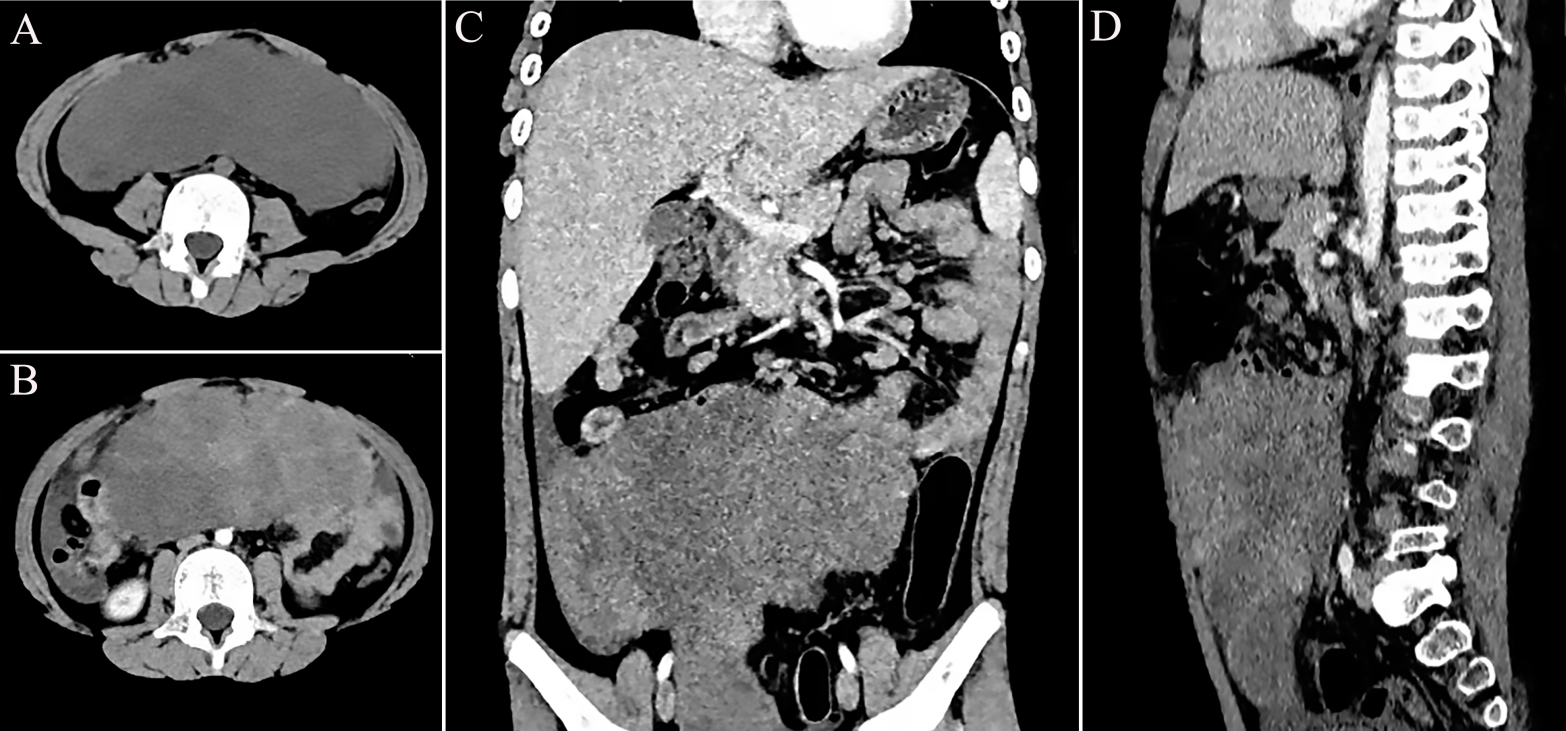
Preoperative imaging presentation of epithelioid inflammatory myofibroblastic sarcoma (EIMS) of the abdominal cavity. **(A)** Computed tomography (CT) of the horizontal position. **(B)** Enhanced CT of the horizontal position. **(C)** Enhanced CT of the coronal position. **(D)** Enhanced CT of the sagittal position.

An 8-year-old boy was admitted to our hospital with a 1-week history of abdominal pain and vomiting. His body mass index (BMI) was measured at 23.17 kg/m^2^. The patient was a student with an unremarkable medical history and no family history of genetic disorders. Physical examination revealed severe abdominal distension accompanied by mild tenderness. The results of the enhanced computed tomography (CT) scan, shown in **Figure 1**, indicated a lower abdominal to pelvic mass measuring approximately 18 × 18 × 6 cm, exhibiting inhomogeneous enhancement. IMT was suspected. The only abnormality noted in the laboratory tests was the platelet count of 766 × 10^9^/L, while the serum tumor markers remained within the normal range.

An ultrasound-guided biopsy of the abdominal mass demonstrated a diffuse infiltrating growth of tumor cells that were round or ovoid in shape, exhibiting cytoplasmic vacuoles and a significant eosinophilic component in the nucleoli. Notably, there was evidence of nuclear pleomorphism, interstitial laxity, and a mucus-like consistency, along with a small degree of acute and chronic inflammatory cell infiltration in the background. Immunohistochemical analysis showed positivity for ALK, with a Ki-67 positivity index of approximately 30%. The expression pattern of ALK was noted to be localized to the nuclear membrane or perinuclear region.

**Figure 2:**
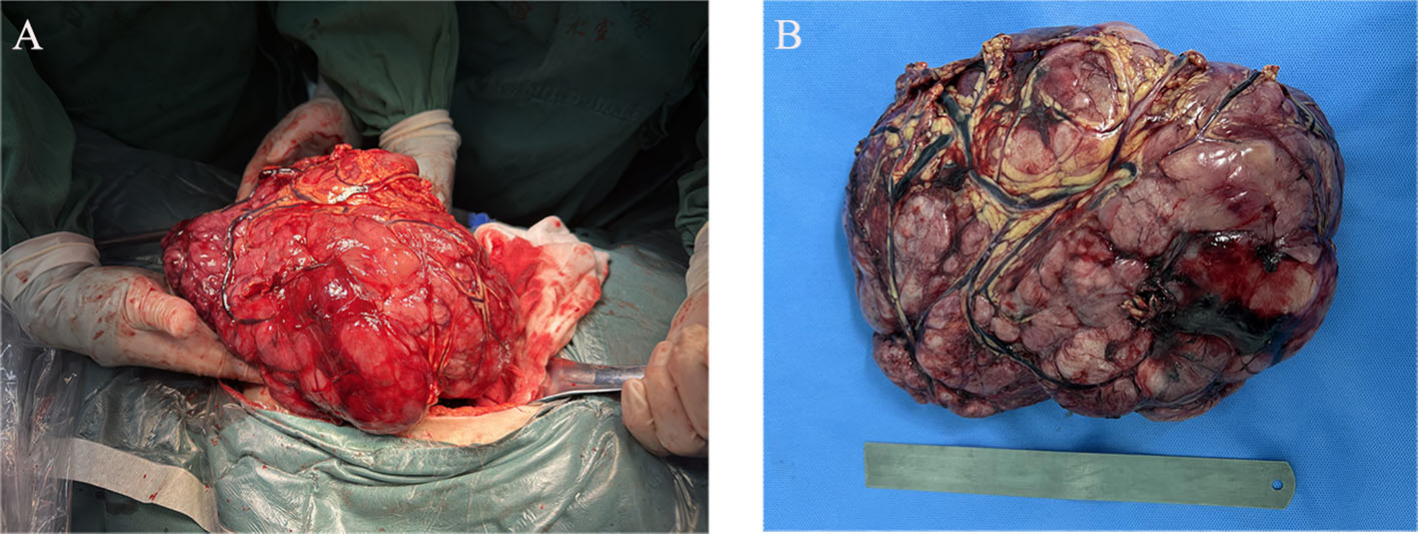
Intraoperative clinical images. **(A)** Abdominal tumor resection. **(B)** Gross examination revealed a large, grayish-red nodular tumor located in the mesentery of the sigmoid colon, measuring 24 × 19 × 9 cm and weighing 2,118 g. The section appeared solid and had a fish-like texture.

**Figure 3:**
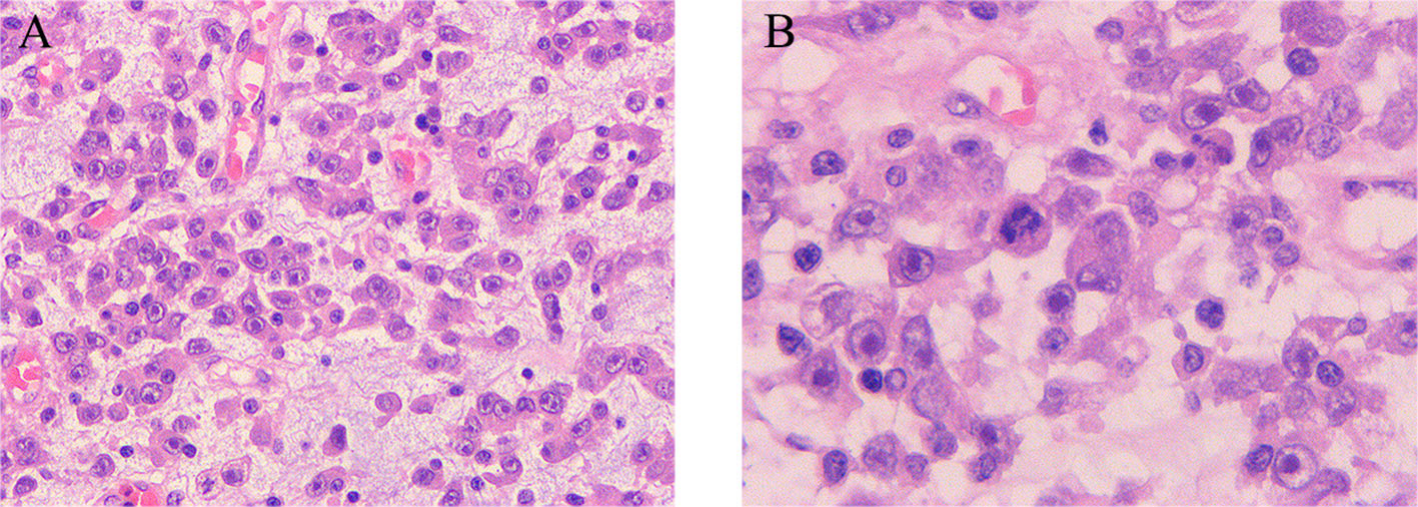
Histopathological manifestations of epithelioid inflammatory myofibroblastic sarcoma. **(A, B)** Diffuse infiltrative growth of tumor cells characterized by epithelioid or rounded morphology, abundant eosinophilic cytoplasm, large nuclei, vacuolated chromatin, pronounced nucleoli, karyomegaly, interstitial laxity, and mucus. Moreover, there is evidence of acute and chronic inflammatory infiltration in the background. **(A, B)** Hematoxylin and eosin (H&E) staining, ×10 original magnification; inserted panel, H&E staining, ×20 original magnification.

Considering that the patient developed incomplete intestinal obstruction during hospitalization, a multidisciplinary team (MDT) discussion led to the decision for the patient to undergo radical tumor resection, as illustrated in the intraoperative clinical views of the intact mass in **Figure 2**. The highest recorded body temperature on the first postoperative day was 37.8°C, with no subsequent fever observed. The volume of fluid drained through the abdominal tube on postoperative day 1 was 70 ml, and the tube was removed on the fourth postoperative day. Postoperative pathology revealed short spindle and elliptical tumor cells, accompanied by a significant infiltration of lymphocytes, plasma cells, and eosinophilic granulocytes within the tumor. The immunohistochemical results, presented in **Figure 3**, indicated ALK (+), desmin (+), Ki-67 (hot spot area approximately 30%), SMA (−), WT-1 (+), D2-40 (+), calretinin (−), CD30 (+), MUC-4 (−), and AAT (NS), which were consistent with EIMS. No tumor infiltration was detected in the surrounding paraneoplastic tissue. In addition, genetic testing performed on this patient confirmed the presence of the *RANBP2*–*ALK* fusion.

The patient was discharged from the hospital 1 week after the operation, having regained the ability to eat normally. At 1 month post-discharge, the patient returned to our hospital due to abdominal distension. An abdominal ultrasound revealed a significant amount of ascites, but the patient’s symptoms of abdominal distension improved following peritoneal puncture and fluid extraction. After 7 months of consistent oral crizotinib treatment (250 mg, twice a day), the patient attended a follow-up appointment at the hospital. During this period of treatment, the patient reported no significant symptoms of nausea, vomiting, abdominal distension, or abdominal pain. The laboratory results were largely normal, and abdominal CT scans indicated that the ascites had completely resolved, with no signs of recurrence. The efficacy of the treatment was assessed as complete remission (CR).

## Discussion

Due to the limited recognition of EIMS, its prevalence remains underestimated, and there are currently no large-scale, convincing clinical trials available. We conducted a review of the previously published literature by searching the PubMed Central, Web of Science, Scopus, Embase, and Google Scholar databases. As of January 1, 2024, only 46 cases of EIMS have been reported in the published literature (5, 10–17).

The site of EIMS is analogous to that of IMT and is most frequently located in the abdominal cavity (11, 18, 19), particularly within the mesentery and greater omentum. However, it can also manifest in other areas, including the thoracic cavity (17, 20, 21), the pelvis (22–24), the oral cavity (25), and the central nervous system (26). Notably, the location of the tumor does not correlate with the age of the patient. A total of 15 cases of pediatric EIMS have been reported, to which we have added one more case. The primary characteristics of the 16 pediatric EIMS cases are summarized in **Table 1**. The mean age of the patients was 7.5 years, with a range from 5 months to 16 years, and 75% of the affected children were boys. Among the cases, 13 children (81%) presented with intra-abdominal EIMS, while one case each was reported from the thoracic and pelvic cavities. The tumor sizes varied from 8 to 22 cm in maximum diameter, with an average of 12.7 cm. Five tumors were multifocal, characterized by a large dominant mass accompanied by multiple small macroscopic nodules in the omentum, mesentery, or peritoneum. Clinically, patients typically present with abdominal pain or a palpable mass, occasionally accompanied by ascites and fever. In our summarized cohort, of the 16 patients, nine were treated with surgery alone or surgery combined with chemotherapy, while seven patients received crizotinib, resulting in partial or complete remission.

**Table 1:** Clinical features of 16 cases of EIMS.

Case	Age	Sex	Site	Size	Symptom	Multifocal	Treatment	Specific drug names of ALK-TKIs	Response at TKIs	PFS at TKIs	Follow-up
1 (27)	2	M	Retroperitoneal	11	Abdominal mass	N	SE				NED (36M)
2 (2)	6	M	Omentum	10.5	NA	N	SE+CT				AWD (13M)
3 (2)	0.7	M	Peritoneum	10	NA	N	SE+CT+RT				DOD (36M)
4 (2)	6	M	Omentum	14	NA	N	SE				NA
5 (28)	7	M	Abdominal cavity	NA	Abdominal pain	N	SE+CT				AWD (5M)
6 (28)	0.7	M	Abdominal cavity	11	Abdominal distention	N	SE+CT				AWD (5M)
7 (29)	8	M	Abdominal cavity	11	High fever	NA	SE				DOD (8M)
8 (1)	16	F	Lung	8	NA	NA	SE+CT+RT+ALKi	Crizotinib	PR	19 (AWD)	AWD (48M)
9 (4)	15	F	Ovary	NA	Abdominal pain	Y	SE+ALKi +CT	Crizotinib/ceritinib	PR	NA	AWD (24M)
10 (30)	14.7	M	Pelvic cavity	NA	Ascites, pleural effusions	N	SE+ALKi	Crizotinib→crizotinib	CR→PR	5→17	AWD (72M)
11 (30)	11.3	M	Abdominal cavity	NA	NA	Y	SE+ALKi	Crizotinib	PR	48 (NED)	NED (48M)
12 (30)	9.1	M	Abdominal cavity	NA	Massive ascites, abdominal compartment syndrome	Y	ALKi→ALKi	Crizotinib→ceritinib+CT	CR→PR	11→6	DOD (11M)
13 (30)	1.4	F	Abdominal cavity	NA	NA	Y	ALKi+SE	Crizotinib	CR	11 (AWD)	NED (9M)
14 (16)	0.4	F	Abdominal cavity	11.4	Abdominal mass	N	SE				NED (6M)
15 (31)	14	M	Retroperitoneal	18	Abdominal pain	Y	SE				DOD (3M)
Current case	8	M	Abdominal cavity	22	Abdominal distention	N	ALKi	Crizotinib	CR	7 (AWD)	AWD (7M)

*F*, female; *M*, male; *Y*, yes; *N*, no; *M*, month; *NA*, data not available; *SE*, surgical excision; *PFS*, progression-free survival; *ALKi*, ALK inhibitor; *CT*, chemotherapy; *RT*, radiation therapy; *CR*, complete response; *PR*, partial response; *NED*, no evidence of disease; *AWD*, alive with disease; *DOD*, death of disease.

EIMS exhibits several common features: 1) epithelioid tumor cells characterized by round nuclei; 2) a mucus-like stroma accompanied by inflammatory cell infiltration; 3) ALK positivity demonstrated by nuclear membrane or perinuclear staining patterns; 4) positive immunostaining for junctional proteins present in the cytoplasm of all tumor cells; and 5) a frequent occurrence of the *RANBP2*–*ALK* fusion genes. All of these features were observed in our case report.

Differential diagnosis of EIMS presents numerous challenges due to the distinct rounded morphology of epithelial cells and the atypical nuclear characteristics. Although ALK expression is essential for diagnosis, it is not specific. EIMS must be differentiated from several conditions, as follows:

1. Anaplastic large cell lymphoma (ALCL): in the rare sarcomatoid variant of ALCL, which demonstrates spindle cell morphology along with positive *CD30*–*ALK* and smooth muscle actin staining, differentiation becomes particularly challenging. To date, no *RANBP2*–*ALK* fusion protein has been identified in ALCL.
2. Malignant mesothelioma: typically shows positive staining for MC, CK5, and calcium-binding protein, but is negative for ALK.
3. Gastrointestinal stromal tumors (GISTs): exhibit positive immunohistochemical staining for CD117, DOG-1, CD34, and C-KIT, but are negative for ALK. In the case of epithelioid GISTs, the nuclei are smaller than those seen in EIMS, and inflammatory cells are absent.
4. Vesicular rhabdomyosarcoma: often ALK-positive, but lacks fibrovascular stroma. The presence of antibodies to myogenin and MyoD aids in the diagnosis.
5. Epithelioid smooth muscle sarcoma: frequently displays cellular heterogeneity and pleomorphism, but does not possess the mucus-like stroma, inflammatory infiltrate, or ALK expression characteristic of EIMS.

Adjuvant chemotherapy and radiotherapy have minimal influence on disease control, making surgical resection the preferred treatment for IMT and extraintestinal manifestations of sarcoidosis (EIMS). Incomplete tumor resection is linked to a significantly increased risk of recurrence. Furthermore, the effectiveness of second-line treatments, including non-steroidal anti-inflammatory drugs, high-dose corticosteroids, biologics, chemotherapy, and radiotherapy, remains uncertain (32).

ALK, first identified in 1994 in the AMS3 cell line derived from anaplastic large cell lymphoma, is a transmembrane protein composed of 1,620 amino acids and is a member of the insulin receptor family. The binding of ligands to ALK results in the activation of several downstream signaling pathways, including the JAK-STAT, RAS-MAPK, PI3K-MTOR, and JUN pathways (33).

The ALK signaling pathway plays a crucial role in the regulation of cell growth, differentiation, and transformation (34). ALK translocations are prevalent in various cancers, primarily resulting in the constitutive activation of ALK kinase activity. Tumors harboring ALK rearrangements, such as non-small cell lung cancer (NSCLC) and IMT, exhibit sensitivity to ALK inhibitors. Recent findings have indicated that ALK inhibitors, including crizotinib, are effective in treating these malignancies (30, 35). Crizotinib is a potent ATP-competitive ALK inhibitor with demonstrated activity against ALK-related cancers, such as ALCL and NSCLC (36, 37). In our case, the patient, who tested positive for ALK, was treated with crizotinib following surgery, and currently, there is no evidence of disease progression. This outcome suggests a favorable response to crizotinib and underscores the importance of this fusion as a target in EIMS carcinogenesis.

Although the ALK inhibitor crizotinib has been reported in the literature to treat EIMS with some success (9, 38), it is primarily utilized as a temporary adjuvant in the management of EIMS. However, prolonged use of crizotinib is associated with several adverse effects, including hematological toxicity, hepatotoxicity, interstitial pneumonitis, prolongation of the QT interval, bradycardia, loss of vision, and tumor lysis syndrome (TLS). Numerous studies have indicated that resistance to the first-generation ALK inhibitor crizotinib typically develops after approximately 1 year of treatment, and it is believed that this resistance may be linked to secondary mutations in the *ALK* gene (39–42). A significant challenge remains in preventing the emergence of drug resistance to ALK inhibitors, necessitating further investigation among patients with EIMS. In addition, the expression of programmed death ligand 1 (PD-L1) in tumor cells is regarded as a predictive marker for the response of the tumor to immunomodulatory therapies that target the programmed cell death 1 (PD-1)/PD-L1 pathway (43), with PD-L1 staining being diffusely positive in cases of EIMS (32). The selection of therapeutic strategies and endpoints following resistance to targeted therapy requires further exploration.

## Conclusion

In conclusion, we present a typical case of EIMS characterized by round or epithelioid cellular morphology, which is associated with a high rate of recurrence and a poor prognosis. To the best of our knowledge, this report represents one of the largest documented cases of EIMS, providing a detailed account of the clinical management and outcomes of the patient while also reviewing the pathological and genetic characteristics of the tumor. The patient demonstrated sensitivity to crizotinib. The identification of EIMS in this instance has significant implications for clinical management as ALK inhibitors constitute an important and potentially effective component of EIMS therapy. Nevertheless, further studies are necessary to investigate the benefits and risks of ALK-targeted therapy in pediatric cases of EIMS.

## Data Availability

The original contributions presented in the study are included in the article/supplementary material. Further inquiries can be directed to the corresponding authors.
